# Effect of beraprost on pulmonary hypertension due to left ventricular systolic dysfunction

**DOI:** 10.1097/MD.0000000000014965

**Published:** 2019-04-19

**Authors:** Li Wang, Xinyi Zhu, Liang-Ping Zhao, Maosong Wang, Xiang Liu, Yuqi Chen, JianChang Chen, WeiTing Xu

**Affiliations:** a Department of Cardiology, The Second Affiliated Hospital of Soochow University; b Emergency Department, The Seventh People's Hospital of Suzhou, China.

**Keywords:** beraprost, heart failure with reduced ejection fraction, prognosis, pulmonary hypertension

## Abstract

Beraprost is used to treat peripheral chronic arterial occlusive disease. However, the efficacy and safety of beraprost in patients with pulmonary hypertension (PH) due to left ventricular systolic dysfunction (PH-HFrEF) remains unknown. The primary objective of this study was to determine the effects of beraprost on PH-HFrEF.

We prospectively recruited patients with PH-HFrEF as determined by echocardiography and right cardiac catheterization. Beraprost sodium was given orally (1 μg/kg/d) added to the usual treatment, and patients were evaluated at 1-year follow-up.

Twenty-five patients were recruited with baseline systolic pulmonary artery pressure (PAP) of 49.5 ± 10.8 mm Hg. Systolic PAP results at 3, 6, 9, and 12 months were 39.1 ± 8.1, 30.4 ± 5.2, 27.7 ± 3.0, and 27.0 ± 4.7 mm Hg, respectively, which were all significantly lower than systolic PAP at baseline (*P* < .05). Left ventricular ejection fraction results at 6 months (43.5 ± 7.0%), 9 months (47.0 ± 5.5%), and 12 months (48.2 ± 4.8%) were significantly higher than at baseline (34.7 ± 9.2%) (*P* < .05). Six-minute walking distance at 3 months (282.8 ± 80.6 m), 6 months (367.1 ± 81.2 m), 9 months (389.8 ± 87.1 m), and 12 months (395.7 ± 83.4 m) increased with time, and all were significantly higher than baseline (190.1 ± 75.5 m) (*P* < .05). One patient developed atrial fibrillation and recovered to sinus rhythm after intravenous administration of amiodarone. There were no instances of cardiac-related death, severe bleeding, or severe impairment of liver function.

Routine oral administration of beraprost sodium added to the usual treatment may improve cardiopulmonary hemodynamics and exercise capacityin patients with PH-HFrEF.

## 1 Introduction

Pulmonary hypertension (PH) is a medically refractory disease with high cost and poor prognosis that includes 5 groups according to clinical presentation, pathological findings, hemodynamic characteristics, and treatment strategies.^[1]^ PH due to left ventricular systolic dysfunction (PH-HFrEF) is common, with high rates of morbidity and mortality.^[2,3]^ PH-HFrEF is characterized by backwards transmission of filling pressures due to impaired left ventricular systolic function with increased pulmonary artery wedge pressure (PAWP), which may further lead to right ventricle overload and right ventricular failure.^[4]^ At present, treatments for pulmonary artery pressure (PAP) of PH-HFrEF patients are lacking.

Beraprost is the first chemically stable and orally active prostacyclin analog, and has antiplatelet and vasodilatory effects. It is mainly used to improve intermittent claudication, pain, and cold symptoms caused by peripheral chronic arterial occlusive disease.^[5]^ A small study including patients with severe primary pulmonary hypertension showed that oral administration of beraprost might result in long-lasting clinical and hemodynamic improvements.^[6]^ Two other studies of patients with primary pulmonary hypertension demonstrated that beraprost improved exercise capacity for up to 3 to 6 months. However, no hemodynamic improvements or long-term outcome benefits were observed.^[7,8]^

Pathogenesis of PH-HFrEF is completely different from that of primary pulmonary hypertension, necessitating different therapeutic approaches. Few studies have evaluated use of beraprost in patients with PH-HFrEF. In this study, we administered beraprost sodium orally to patients with PH-HFrEF on a routine basis to investigate the efficacy and safety of this therapy.

## 2 Methods

### 2.1 Study subjects

This was a prospective pilot study conducted in an Asian country. We recruited consecutive patients hospitalized at the Second Affiliated Hospital of Soochow University China with a diagnosis of heart failure. Patients were examined by echocardiography to determine whether there was left ventricular systolic dysfunction (left ventricular ejection fraction [LVEF] <45%) and PH (systolic PAP >40 mm Hg). Patients with shock, hemorrhagic disease, severe hepatic insufficiency, acute stage myocardial infarction, pregnancy, malignant tumor, and other types of pulmonary hypertension (idiopathic, connective tissue diseases, drugs, thromboembolic, chronic lung disease, portal hypertension, congenital heart disease, etc) were excluded. Details of the study protocol were explained to the eligible patients and informed consent was obtained. The study protocol was approved by the Medical Ethics Committee of the Second Affiliated Hospital of Soochow University.

### 2.2 Coronary angiography and right heart catheterization

Coronary angiography and right heart catheterization were performed using standard techniques for patients who were eligible for inclusion. Coronary angiography was used to determine whether patients had coronary heart disease. Right heart catheterization was used to measure mean PAP and PAWP. Inclusion criteria included mean PAP ≥25 mm Hg and PAWP >15 mm Hg.

### 2.3 Laboratory investigations and clinical data collection

NT-proBNP, white blood cells, hemoglobin, platelets, fasting plasma glucose, blood urea nitrogen, creatinine, and serum lipid profiles including triglyceride, total cholesterol, low-density lipoprotein cholesterol (LDL-C), and high-density lipoprotein cholesterol (HDL-C) were assessed using standard methods. Demographic and clinical characteristics of the recruited patients were collected from hospital case records and included age, sex, cigarette smoking status, hypertension, diabetes mellitus, principal diagnosis, and so on. Patient height (m) and weight (kg) in light clothing was measured, and body mass index (kg/m^2^) was calculated.

### 2.4 Drug use scheme

Eligible patients with PH-HFrEF were given beraprost sodium orally (1 μg/kg/d across 3 administrations) on a routine basis. Patients were also taking various other medications including diuretics, spironolactone, angiotensin-converting enzyme inhibitors (ACEIs), angiotensin receptor blockers (ARBs), β-blockers, digoxin, and other drugs. If no complications, such as shock, severe bleeding, or severe liver function damage occurred, the dosage of beraprost sodium was unchanged.

### 2.5 Six-minute walk test

The 6-minute walk test was given to the recruited patients at the time of selection, and at 3, 6, 9, and 12-month follow-up. Six-minute walk distance (6MWD) was recorded. Patients were accompanied by a doctor for the entirety of the test, and rescue medicine and equipment were readily available.

### 2.6 Echocardiographic assessment

Echocardiography was performed at case admission, and at 3, 6, 9, and 12-month follow-up using a Vivid 7 ultrasound system with a standard imaging transducer. All measurements were performed following the recommendations of the American Society of Echocardiography.^[9]^ Right ventricular (RV) systolic pressure is equal to systolic PAP in the absence of pulmonary stenosis. Systolic PAP is equal to the sum of the right atrial (RA) pressure and the RV-to-RA pressure gradient during systole. RA pressure was estimated based on the echocardiographic features of the inferior vena cava and assigned a standard value.^[10]^ The RV-to-RA pressure gradient was calculated as 4Vt^2^ using the modified Bernoulli equation, where Vt is the velocity of the tricuspid regurgitation jet in m/s.

### 2.7 Endpoints and adverse reactions

The recruited patients were re-evaluated at least once every 3 months. Occurrences of major adverse cardiac events (MACE), such as cardiac death, heart failure readmission, nonfatal myocardial infarction, and new onset of atrial fibrillation, were recorded.

Adverse drug reactions including shock, severe bleeding (cerebral hemorrhage, gastrointestinal bleeding, pulmonary hemorrhage, etc), and severe liver function damage were recorded during the follow-up period.

### 2.8 Statistical analyses

Exploratory analyses involving quantitative data were performed with independent-sample *t* tests (or nonparametric Wilcoxon-Mann-Whitney tests if the data were non-normally distributed). Data were exported to SPSS 17.0 for Windows (SPSS Inc., Chicago, IL) for analysis, and all statistical tests were conducted at the 5% level of significance.

## 3 Results

### 3.1 Baseline characteristics

Between January, 2016 and December, 2016, 25 eligible PH-HFrEF patients, including 17 men and 8 women with an average age of 68.9 ± 7.6 years, were recruited and followed for 12 months. The hospitalization duration at recruitment was 8.6 ± 2.5 days. Baseline demographics, clinical data, and blood and echocardiographic characteristics of the study patients are summarized in Table 1. Coronary angiography showed that 15 patients had ischemic heart disease and 10 patients had dilated cardiomyopathy and hypertensive heart disease. Right heart catheterization showed that systolic PAP of the study patients was 48.7 ± 9.4 mm Hg, mean PAP was 30.1 ± 5.2 mm Hg, and PAWP was 20.3 ± 4.6 mm Hg. Baseline LVEF and systolic PAP as determined by echocardiography were 34.7 ± 9.2% and 49.5 ± 10.8 mm Hg, respectively.

**Table 1:**
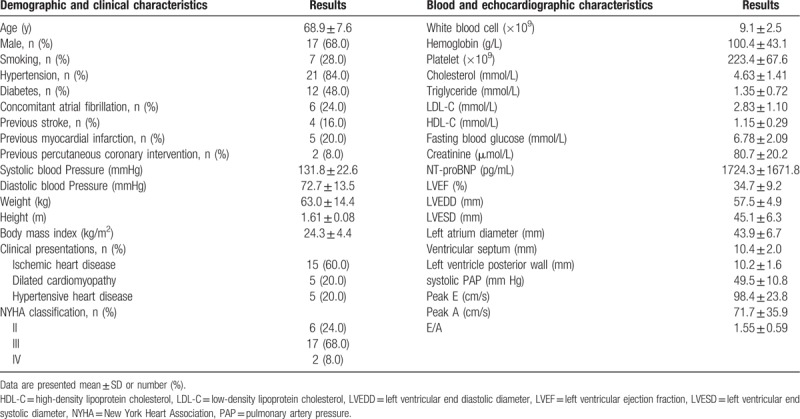
Demographic, clinical, blood and echocardiographic characteristics.

### 3.2 Medications

The recruited patients were given beraprost sodium orally (1 μg/kg/d across 3 administrations) for 1 year. The dosage of beraprost sodium was unchanged since there were no occurrences of shock, severe bleeding, or severe liver dysfunction. The percentage of patients receiving β-blockers, spironolactone, or ACEIs/ARBs was greater than 60%. Nine (36%) patients underwent interventional coronary artery revascularization (Table 2).

**Table 2:**
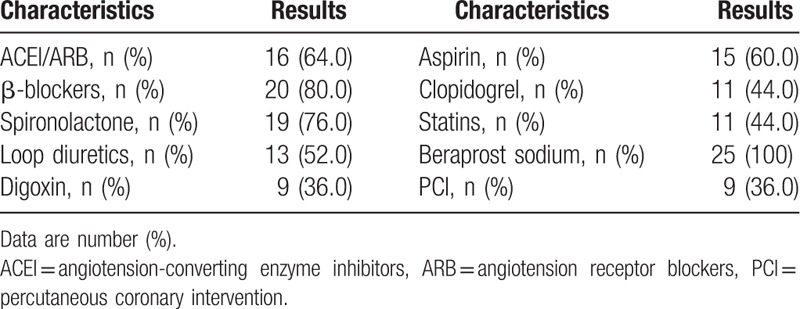
Medications and interventions.

### 3.3 Change in pulmonary artery pressure

**Figure 1:**
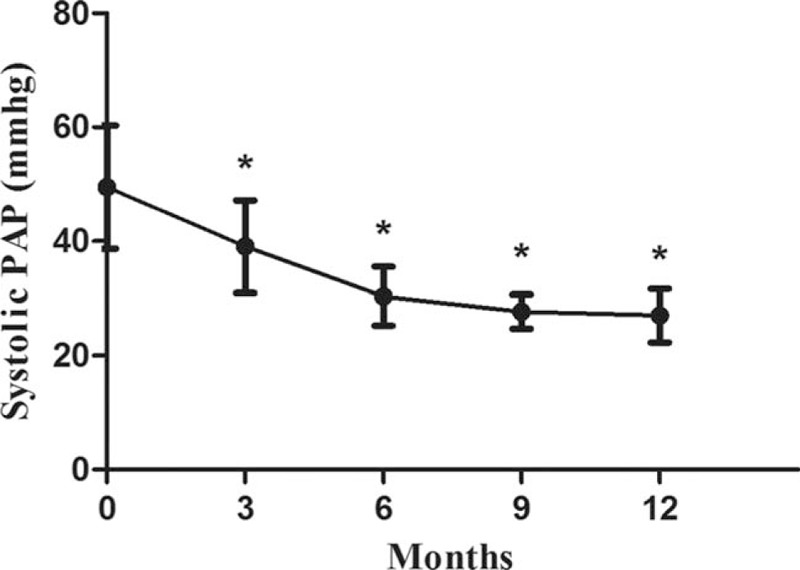
Change in systolic pulmonary artery pressure from baseline to 12 months. Values are expressed as mean ± SD. “^∗^” represents *P* < .05 compared with baseline (month 0). PAP = pulmonary artery pressure.

Systolic PAP measurements by echocardiography at 3, 6, 9, and 12-month follow-up were 39.1 ± 8.1, 30.4 ± 5.2, 27.7 ± 3.0, and 27.0 ± 4.7 mm Hg, respectively, which were all significantly lower than at baseline (49.5 ± 10.8 mm Hg), and showed a downward trend (*P* < .05) (Fig. 1).

### 3.4 Change in left ventricular ejection fraction

**Figure 2:**
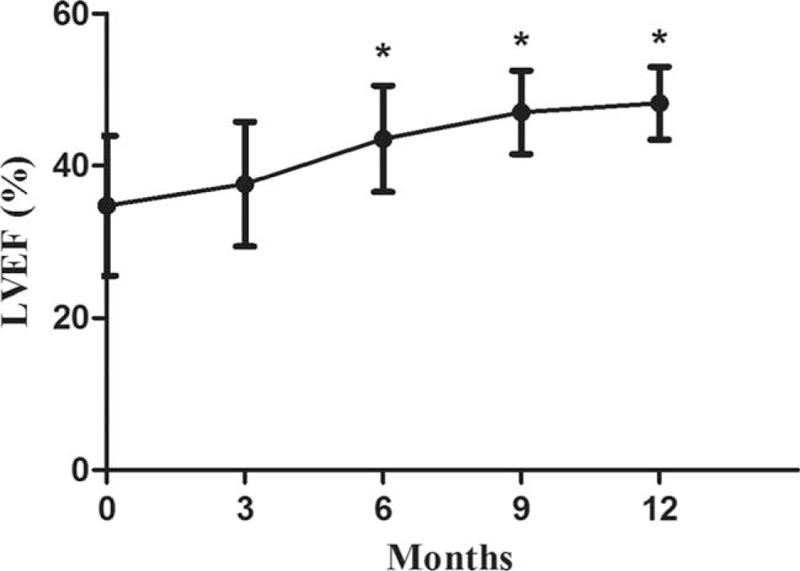
Change in left ventricular ejection fraction from baseline to 12 months. Values are expressed as mean ± SD. “^∗^” represents *P* < .05 compared with baseline (month 0). LVEF = left ventricular ejection fraction.

Left ventricular ejection fraction at 6 months (43.5 ± 7.0%), 9 months (47.0 ± 5.5%), and 12 months (48.2 ± 4.8%) was significantly higher at each point than at baseline (34.7 ± 9.2%) (*P* < .05). LVEF at 3 months was 37.6 ± 8.2%, which was not significantly different from baseline. After a year of treatment, the LVEF was generally increasing (Fig. 2).

### 3.5 Change in 6-minute walk distance

**Figure 3:**
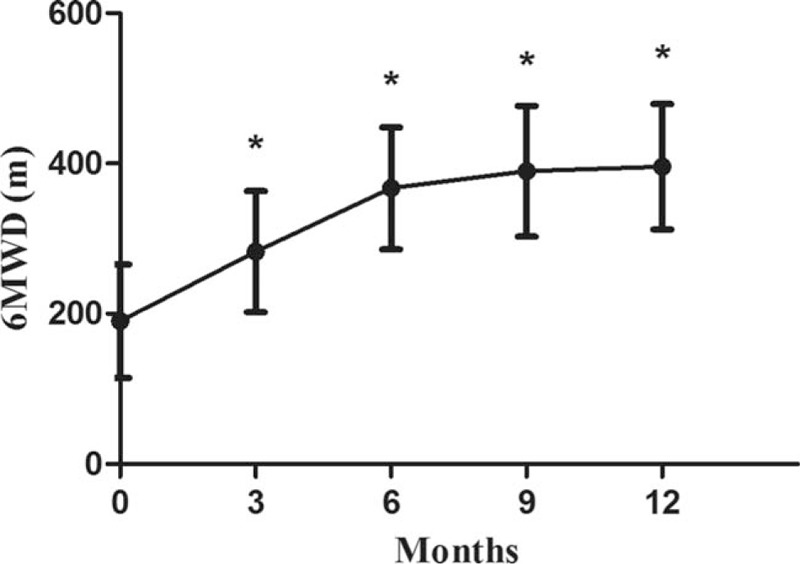
Change in 6-minute walk distance from baseline to 12 months. Values are expressed as mean ± SD. “^∗^” represents *P* < .05 compared with baseline (month 0). 6MWD = 6-minute walk distance.

The 6MWD at 3 months (282.8 ± 80.6 m), 6 months (367.1 ± 81.2 m), 9 months (389.8 ± 87.1 m), and 12 months (395.7 ± 83.4 m) months increased gradually, and all distances were significantly higher than the baseline value (190.1 ± 75.5 m) (*P* < .05) (Fig. 3).

### 3.6 Clinical outcomes

One patient experienced new onset of atrial fibrillation, and recovered to sinus rhythm after intravenous administration of amiodarone. There were no instances of cardiac death, heart failure readmission, or myocardial infarction during the follow-up period. Two patients with persistent atrial fibrillation at admission spontaneously converted to sinus rhythm without using special antiarrhythmic drugs.

During the follow-up period 1 patient had hematochezia and was treated with clopidogrel and aspirin after coronary stenting. A hemorrhoid was found, and the presence of tumors was negative as determined by colonoscopy. Hematochezia recovered within 3 days of discontinuation of aspirin. One week after recovery from hematochezia, aspirin was administered with longer intervals, and hematochezia did not recur. There were no occurrences of severe bleeding (cerebral hemorrhage, gastrointestinal bleeding, pulmonary hemorrhage, etc) or severe impairment of liver function.

## 4 Discussion

This pilot study prospectively evaluated the efficacy and safety of beraprost in patients with PH-HFrEF. The results showed that routine oral administration of beraprost sodium added to the usual treatment reduced systolic PAP, and improved LVEF and 6MWD. There were no instances of cardiac death, myocardial infarction, shock, severe bleeding, or severe impairment of liver function, which indicated good efficacy and safety of beraprost in these patients.

Pulmonary hypertension due to left ventricular systolic dysfunction is different from other types of PH with regard to pathogenesis and management. Backwards transmission of filling pressures due to impaired left ventricular systolic function is the main cause of PH-HFrEF, along with high PAWP. Unlike heterozygous BMPR2 mutations, which account for approximately 75% of familial pulmonary arterial hypertension cases, no specific genetic linkage has been identified for PH-HFrEF.^[2,11]^ PH is prevalent in patients with chronic heart failure. Up to 60% of patients with severe left ventricular systolic dysfunction may also have PH, and almost all patients with severe symptomatic mitral valve disease have PH with or without heart failure.^[12,13]^ Despite appropriate treatment, patients with PH-HFrEF still have a high 5-year readmission rate and all-cause mortality rate.^[14]^

Management of PH-HFrEF is a systematic process. Treatment of group 1 PH (idiopathic, heritable, drug or toxin-induced, associated with connective tissue disease, etc) in the ESC/ERS guidelines does not apply to patients with PH-HFrEF.^[1]^ Most clinical findings showed that PH-specific therapies (eg, endothelin receptor antagonists, phosphodiesterase type 5 inhibitors, soluble guanylyl cyclase stimulators) had no effect on PH-HFrEF, and some may even be harmful.^[15–17]^ General therapies for heart failure including diuretics, digoxin, ACEIs, and β-blockers are recommended for treatment of PH-HFrEF.^[18]^ Diuretics are the main medical treatment for relief of fluid load and congestion, and ACEIs and β-blockers improve prognosis. Furthermore, identifying and treating possible causes of heart failure, such as coronary artery disease, cardiomyopathy, and heart valve disease, is important.^[19]^

Whether prostacyclin and its analogs can be used in patients with heart failure is controversial. Several studies of intravenous prostacyclin in patients with left ventricular systolic dysfunction after cardiac surgery showed that prostacyclin could decrease mean PAP, PAWP, and systemic and pulmonary vascular resistances, and increased cardiac output was comparable to that resulting from sodium nitroprusside or inhaled nitric oxide treatment.^[20,21]^ In animal models and human trials, prostacyclin decreased PAP and improved function of the right heart in right ventricular failure because of its vasodilatory effects.^[22,23]^ However, a randomized controlled trial of epoprostenol infusion for treatment of severe congestive heart failure yielded different results.^[24]^ This trial was terminated early because of a strong trend of decreased survival in patients treated with epoprostenol. Intravenous epoprostenol was not associated with improvement in 6MWD or quality of life, but was associated with increased risk of death.^[24]^

Beraprost is a stable, orally administered prostacyclin analog recommended for treatment of chronic peripheral arterial disease.^[5]^ Like prostacyclin, beraprost acts on the prostacyclin receptor in platelets and vascular smooth muscle, activates adenylate cyclase, increases intracellular cyclic adenosine monophosphate concentration, inhibits Ca2+ influx and thromboxane A2 production, and thus induces antiplatelet and vasodilatory effects. In addition, beraprost can increase endothelial nitric oxide synthase expression and nitric oxide production in murine and bovine aortic endothelial cells,^[25]^ and suppress TNF-α expression in human monocytes via mitogen-activated protein kinase pathways,^[26]^ all of which improve vascular diseases. Previous studies of beraprost for treatment of pulmonary hypertension evaluated group 1 PH in the ESC/ERS guidelines.^[6–8]^ Although a small study with 13 patients with severe pulmonary arterial hypertension demonstrated that beraprost may result in long-lasting clinical and hemodynamic improvements,^[6]^ 2 randomized controlled trials with more than 100 patients with pulmonary arterial hypertension confirmed that beraprost only improved exercise capacity, which persisted for up to 3 to 6 months, but did not improve long-term prognosis.^[7,8]^ No obvious side effects were observed.

Pulmonary hypertension due to left ventricular systolic dysfunction is completely different from group 1 and other types of PH in both pathogenesis and treatment. This study was the first to use beraprost in patients with PH-HFrEF, regardless of dosage and duration. Based on beneficial effects of beraprost on vessel dilatation and antiplatelet activity, routine oral beraprost treatment was hypothesized to provide therapeutic benefit to patients with PH-HFrEF, which was confirmed in this pilot study. Several studies have shown that beraprost can inhibit myocardial apoptosis and myocardial fibrosis, which is 1 of the possible intrinsic mechanisms for beraprost improving the prognosis of PH-HFrEF patients.^[27,28]^

Major side effects of beraprost include severe bleeding, hypotension, and shock. In patients with PH-HFrEF, therapeutic strategies typically include combinations of diuretics, ACEIs, and antiplatelet drugs, which may increase the incidence of side effects. Therefore, when side effects occur, it is important to evaluate which drugs are responsible. Moreover, follow-up frequency should be increased. Once the cause of side effects is discovered, the type and dosage of the drugs should be adjusted accordingly. In this study, beraprost sodium was administered at 1 μg/kg/d and was not titrated to a larger dose. If bleeding or shock had occurred, the cause would have been investigated, and the dosage of beraprost sodium or other drugs would have been adjusted. In this study, a patient experienced hematochezia, which was caused by hemorrhoids. After adjusting the aspirin dosing interval, hematochezia stopped.

### 4.1 Limitations

Our study had the following limitations. The small sample size precluded making conclusive statements regarding our results. In addition, follow-up PAP results obtained by echocardiography were not as accurate as right cardiac catheterization. Furthermore, this study was an observational study, and no control group was evaluated. As such, more randomized controlled studies are needed to investigate the efficacy and safety of beraprost in patients with PH-HFrEF.

### 4.2 Future directions

A multicenter randomized controlled clinical trial (ChiCTR-IPR-17012961) with larger samples designed based on the results of this pilot study is in progress and we will detect the dynamic changes of NT-proBNP in this study. In the near future, we will have more evidence for clinicians.

## 5 Conclusions

Routine oral administration of beraprost sodium added to the usual treatment may further decrease PAP, and improve LVEF and 6MWD in patients with PH-HFrEF, indicating cardiopulmonary hemodynamic and exercise capacity benefits. Meanwhile, treatment with beraprost exhibited good safety.

## Acknowledgments

The authors gratefully acknowledge the assistance of Miss Yan-Ni Wu and Jing Zhu with patient recruitment.

## Author contributions

**Conceptualization:** Yuqi Chen.

**Data curation:** Xinyi Zhu, Yuqi Chen.

**Formal analysis:** Xinyi Zhu.

**Funding acquisition:** Liang-Ping Zhao.

**Investigation:** Maosong Wang, Xiang Liu.

**Methodology:** Xinyi Zhu, Maosong Wang, Xiang Liu.

**Project administration:** WeiTing Xu.

**Supervision:** JiangChang Chen, WeiTing Xu.

**Validation:** Maosong Wang, Yuqi Chen.

**Writing – original draft:** Li Wang.

**Writing – review & editing:** Liang-Ping Zhao.
